# Use of surface‐guided radiation therapy in combination with IGRT for setup and intrafraction motion monitoring during stereotactic body radiation therapy treatments of the lung and abdomen

**DOI:** 10.1002/acm2.12852

**Published:** 2020-03-20

**Authors:** John H. Heinzerling, Carnell J. Hampton, Myra Robinson, Megan Bright, Benjamin J Moeller, Justin Ruiz, Roshan Prabhu, Stuart H. Burri, Ryan D. Foster

**Affiliations:** ^1^ Levine Cancer Institute Southeast Radiation Oncology Group Atrium Health Charlotte NC USA; ^2^ Levine Cancer Institute Department of Radiation Oncology Atrium Health Charlotte NC USA; ^3^ Levine Cancer Institute Department of Biostatistics Atrium Health Charlotte NC USA

**Keywords:** IGRT, intrafraction motion, localization, SBRT, SGRT

## Abstract

**Background and purpose:**

Multiple techniques can be used to assist with more accurate patient setup and monitoring during Stereotactic body radiation therapy (SBRT) treatment. This study analyzes the accuracy of 3D surface mapping with Surface‐guided radiation therapy (SGRT) in detecting interfraction setup error and intrafraction motion during SBRT treatments of the lung and abdomen.

**Materials and Methods:**

Seventy‐one patients with 85 malignant thoracic or abdominal tumors treated with SBRT were analyzed. For initial patient setup, an alternating scheme of kV/kV imaging or SGRT was followed by cone beam computed tomography (CBCT) for more accurate tumor volumetric localization. The CBCT six degree shifts after initial setup with each method were recorded to assess interfraction setup error. Patients were then monitored continuously with SGRT during treatment. If an intrafractional shift in any direction >2 mm for longer than 2 sec was detected by SGRT, then CBCT was repeated and the recorded deltas were compared to those detected by SGRT.

**Results:**

Interfractional shifts after SGRT setup and CBCT were small in all directions with mean values of <5 mm and < 0.5 degrees in all directions. Additionally, 25 patients had detected intrafraction motion by SGRT during a total of 34 fractions. This resulted in 25 (73.5%) additional shifts of at least 2 mm on subsequent CBCT. When comparing the average vector detected shift by SGRT to the resulting vector shift on subsequent CBCT, no significant difference was found between the two.

**Conclusions:**

Surface‐guided radiation therapy provides initial setup within 5 mm for patients treated with SBRT and can be used in place of skin marks or planar kV imaging prior to CBCT. In addition, continuous monitoring with SGRT during treatment was valuable in detecting potentially clinically meaningful intrafraction motion and was comparable in magnitude to shifts from additional CBCT scans. PTV margin reduction may be feasible for SBRT in the lung and abdomen when using SGRT for continuous patient monitoring during treatment.

## 1 INTRODUCTION

Stereotactic body radiation therapy (SBRT) has now become standard treatment for early stage inoperable nonsmall cell lung cancer and is increasingly used for treatment of other primary tumors as well as oligometastatic disease.^1^ Stereotactic body radiation therapy includes use of high dose per fraction treatments over few fractions with sharp dose gradients necessitating precision dose delivery for each treatment. Typically, image‐guided radiation therapy (IGRT) is a required part of daily SBRT patient setup and is most commonly performed with integrated cone beam computed tomography (CBCT) that allows accurate target localization prior to treatment.^2^, ^3^ Most IGRT systems, however, do not manage potential patient displacement during the entire treatment, otherwise referred to as “intrafraction motion.” IGRT systems that do address intrafraction motion often involve placement of fiducials or electromagnetic transponders that require invasive procedures and require use of ionizing radiation that can increase treatment time or radiation exposure to the patient.^4^, ^5^

Surface‐guided radiation therapy (SGRT) is a technique that uses nonionizing visible light directed at a patient setup in the treatment position to create a 3D surface rendering that can be matched to the reference surface of the patient. Surface‐guided radiation therapy has been validated to help with patient setup prior to the initiation of treatment in breast and other cancers,^6^, ^7^ but most of these studies involved comparison of setup based on portal imaging of large fields not typically used in stereotactic treatments or have used phantom systems for validation of accuracy.^8^ Surface‐guided radiation therapy can also be utilized to monitor the surface of patients continuously during treatment to detect intrafraction patient displacement. This method of intrafraction motion monitoring does not include additional use of ionizing radiation or invasive placement of markers for tumor tracking. Surface‐guided radiation therapy monitoring during treatment has been used successfully in the clinical setting including for deep inspiration breath hold in breast cancer, as well as in patients receiving stereotactic radiosurgery to the brain;^8^, ^9^ yet little data exist quantifying the accuracy of SGRT in detecting intrafraction motion of internal targets during SBRT treatments. Some studies have shown good agreement between surface imaging and spirometry^10^, ^11^ and fluoroscopy^12^ although our study does not use SGRT for respiratory motion management. In addition, due to the deformable anatomy in the thorax and abdomen, not much data are available on the reliability of patient setup using SGRT for small targets in these anatomical locations.

The purpose of this study is to first quantify the accuracy of SGRT in positioning of patients prior to CBCT during SBRT treatments in comparison to planar kV imaging; and second, to quantify the accuracy of SGRT in detecting intrafraction motion during SBRT treatments in order to assess the reliability of this system for interfraction motion reduction and intrafraction motion detection to ensure high precision throughout the entire SBRT treatment process.

## 2 MATERIALS AND METHODS

### 2.1 Patients/SBRT treatments

The data for this study were collected under an IRB‐approved retrospective chart review. All patients were simulated and treated using the CIVCO Body ProLok ONE SBRT Immobilization System and vacuum cushion. The ProLok system has been found to be an effective immobilization system for SBRT patients and comparable to other systems.^13^, ^14^, ^15^ All lung and liver patients were treated with abdominal compression for tumor motion management using the abdominal compression plate and underwent 4DCT simulation. SBRT patients were treated with dynamic conformal arcs or RapidArc with either 6 FFF (lung) or 10 FFF (liver) photons on a Varian TrueBeam with a Millennium 120 MLC and all patients were treated on a six degree of freedom couch. To account for tumor motion due to respiration, lung tumor ITVs were contoured on the Maximum Intensity Projection (MIP) 4DCT reconstruction and liver ITVs were contoured on the Minimum Intensity Projection (MinIP) 4DCT reconstruction with standard planning target volume (PTV) margin expansions of 5 mm for lung and abdominal/liver targets. Dose calculation was performed on the average intensity projection (AIP), which was also used as the reference for IGRT matching. The MIP, MinIP, and AIP datasets were reconstructed from all respiratory phases of the 4DCT. No patients had implanted fiducials for matching and all patients were treated free‐breathing, that is, we did not gate the beam based on respiration.

Surface‐guided radiation therapy using the OSMS system has been described previously in detail,^16^ but will be briefly discussed here. The system projected a speckle pattern on the patient using optical light and cameras with three separate camera pods were used to detect the pattern and map the patient surface. As it uses optical light, no additional radiation was given to the patient. In our clinic, the system calibration was checked daily before treatments began and the system was calibrated monthly, including a calibration to the isocenter of the MV treatment beam using radiographic imaging of a phantom. Published results have shown that relative to CBCT, OSMS has an accuracy of ≤±0.25 mm and ± 0.20° when localizing a phantom.^17^ We have found our systems to be accurate to within 0.3 mm and 0.20° during acceptance and commissioning using the Vision RT cube phantom when compared to radiographic image guidance. The system frame rate varied from 1.5 to 3 frames per second, depending on the size of the region of interest that was being tracked. The region of interest used for tracking was superior to the abdominal compression arch and consisted of the patient’s chest and laterally down to the edge of the vacuum cushion used for immobilization.

### 2.2 Study design

#### 2.2.1 SGRT utilization for patient setup prior to CBCT

To first quantify the accuracy of SGRT in patient positioning prior to SBRT treatments compared to orthogonal kV images, patients were initially positioned using either SGRT or planar orthogonal kV images on an alternating schedule prior to CBCT. For each SGRT fraction, patients were initially setup to the DICOM SGRT reference surface that was created from the body contour outlined on the AIP reconstruction CT using a density threshold of 0.6 g/cc (−350 HU). Initial setup was performed using tolerances of 2 mm for translations and 1 degree for rotations and adjustments were made such that offsets due to breathing motion were fluctuating about 0. On kV imaging days, patients were setup initially to skin marks, imaged with orthogonal planar kV images, and then bony anatomy in the target region was used to match the images compared to digitally reconstructed radiographs created from the treatment planning CT scan. After each setup method, CBCT was performed and additional shifts were made to match the internal target position prior to treatment. All CBCTs were evaluated by the treating physician. For lung targets, the gross tumor volume (GTV) was matched with the corresponding reference image and the PTV was evaluated to ensure that the GTV was adequately covered. For liver targets, because the GTV was not usually visible on CBCT, the liver contour was initially matched with the reference image and then the hepatic veins and ligaments in proximity to the internal liver target were evaluated with the reference image to ensure accurate targeting of the area of liver containing the GTV. For adrenal targets, the adrenal gland and GTV were matched to the reference image. For spine targets, the bony anatomy was first matched to the reference image and then adjusted based on the GTV within the bone. The additional shifts from the CBCT were recorded to determine the interfraction setup error from the initial SGRT or kV imaging localization for comparison.

#### 2.2.2 SGRT utilization for patient monitoring during treatment

After the initial CBCT shifts were applied, a new gated reference surface was acquired by the SGRT system and used to continuously monitor the patient during treatment. The gated surface capture collects approximately 30 frames over 6 sec, resulting in a plot of the patient respiratory cycle during those 6 sec. The reference surface for monitoring was chosen from the gated capture to be at the 50% amplitude of the patient’s respiratory cycle. For the intrafraction motion analysis, tolerances for intrafraction motion were defined as 2 mm translations along any axis and 1 degree rotations about any axis with a maximum of 2 sec out of tolerance before an auto beam hold was enacted. The 2 sec tolerance was used to reduce the number of beam holds that were due to the patient’s normal respiratory motion. The beam was held automatically by the SGRT system if the patient was out of tolerance for more than 2 sec and the beam hold remained in effect until the patient went back in tolerance. If a patient’s position as reported by the SGRT system either exceeded these thresholds three times in the fraction or went out of tolerance and stayed out of tolerance, the beam was turned off after 2 sec, the SGRT detected shifts were recorded and a repeat CBCT was performed. To determine the SGRT shifts, several (3–5) report captures were taken using the SGRT software and the average positions from these captures were used for the SGRT shifts. Any shifts that were performed after repeat CBCT with tumor volume matching were recorded as “intrafraction patient motion.” The shifts were then applied and a new SGRT reference image was captured. The physician performed all IGRT matching. The intrafraction shifts detected by SGRT were then compared to those determined on the subsequent CBCT.

### 2.3 Statistics

To determine if SGRT provided comparable accuracy for initial setup of SBRT patients to that provided by kV/kV matching, linear mixed models for repeated measures were used to analyze the rotational and translational shift measurements. Shifts were modeled with main effects for method of setup (kV/kV or SGRT) and temporal treatment period, as well as a lesion‐specific identifier as a random factor to account for within‐lesion correlation. Method was evaluated at α = 0.05 level of significance. Least square mean shifts and standard errors of the mean (SEM) associated with each method were estimated from these models. To investigate the impact of patient‐ or lesion‐level factors on differences in shifts between the two methods, each shift measurement was evaluated with models that included an interaction term between method and factor. Factors included in the evaluated interaction models were those that were significantly associated with shifts, irrespective of method (at α = 0.05 significance level).

To assess the reliability of SGRT in detection and quantification of clinically significant intrafraction motion for SBRT treatments of the lung and abdomen, a paired t‐test was used to compare the difference in vector shift between the SGRT and CBCT methods (at the α = 0.05 significance level). Factors impacting the difference in vector shifts were identified using univariable and multivariable ANOVA models. Patient‐ and lesion‐related factors were analyzed individually with univariable models (at the α = 0.05 significance level). Backward elimination and forward selection were used to identify the factors that were independently associated with vector shift differences (entry/elimination was evaluated at the α = 0.05 level of significance).

## 3 RESULTS

### 3.1 Patient characteristics

A total of 71 patients with 85 malignant thoracic or abdominal tumors treated with SBRT were included in both analyses. Patient characteristics including tumor location, target size, body mass index (BMI), and range of respiratory motion (4D ROM) on 4D CT are seen in Table 1. The majority of patients had lung tumors and respiration induced tumor motion was well controlled with abdominal compression as observed on 4D CT.

**Table 1:** Patient and tumor characteristics.

	*n = 71 subjects*	
Gender, n (%)		
Male	37	52.1%
Female	34	47.9%
Age		
Mean (SD)	71.3	10.6
Median (Range)	73.0	47–87
BMI		
Mean (SD)	26.8	6.2
Median (Range)	26.3	13.8–40.3
BMI Category		
Underweight (BMI < 18.5)	6	8.5%
Normal (18.5 ≤ BMI <25)	22	31.0%
Overweight (25 ≤ BMI <30)	21	29.6%
Obese (BMI ≥ 30)	22	31.0%

	*n = 85 lesions*	
Site, n (%)		
Lung	63	74.1%
Liver	10	11.8%
Adrenal	3	3.5%
Spine	2	2.4%
Other	7	8.2%
Location		
Peripheral	57	67.1%
Central	14	16.5%
N/A	14	16.5%
4D ROM (cm)		
Mean (SD)	0.49	0.30
Median (Range)	0.44	0.00–1.24
PTV volume (cc)		
Mean (SD)	31.8	36.7
Median (Range)	20.3	4.6–218.5
ITV volume (cc)		
Mean (SD)	11.8	18.6
Median (Range)	5.7	0.6–136.0

### 3.2 Comparison of SGRT to kV planar imaging in interfraction positioning during SBRT

For the initial comparison of SGRT to kV planar imaging in interfraction positioning prior to SBRT treatment, 46 patients with 58 total tumors were analyzed for a total of 238 fractions. Table 2 shows the estimated mean magnitude (absolute value) and standard error of the mean of the shift on CBCT after initial positioning with kV/kV imaging vs SGRT in all six degrees of freedom and associated *P*‐values of comparison. Other than the longitudinal direction, there was no significant difference between method of setup and resulting additional shifts on volumetric imaging. Patient and tumor characteristics that were analyzed for impact on setup method included age, tumor site (lung, liver, adrenal, spine, other) tumor location (peripheral vs central), BMI, and 4D ROM. Factors affecting setup methods and corresponding effect modification are shown in Table 3. As indicated by significant interactions, the subject’s BMI, age, 4D ROM, or tumor location may impact the shifts on CBCT with each of the setup methods. All 46 patients and 58 lesions in this part of the analysis were included in the analysis of SGRT for patient monitoring during treatment.

**Table 2:** Mean quantitative shift and standard error of the mean on CBCT after initial positioning with kV/kV imaging vs SGRT.

	Method *P*‐value*	kV/kV Mean Shift (Standard Error)	SGRT Mean Shift (Standard Error)
Vertical shift (cm)	0.291	0.26 (0.03)	0.29 (0.03)
Longitudinal Shift (cm)	0.019	0.28 (0.04)	0.41 (0.04)
Lateral shift (cm)	0.489	0.24 (0.03)	0.22 (0.02)
Translational vector	0.107	0.54 (0.05)	0.63 (0.04)
Pitch (˚)	0.335	0.35 (0.09)	0.44 (0.07)
Roll (˚)	0.587	0.35 (0.09)	0.40 (0.08)
Rotation (˚)	0.204	0.28 (0.08)	0.39 (0.07)
Rotational vector	0.189	0.75 (0.13)	0.93 (0.12)

CBCT, cone beam computed tomography; SGRT, Surface‐guided radiation therapy.

* indicates F‐test for fixed effects.

**Table 3:** Patient and tumor characteristics affecting mean quantitative interfraction shifts by setup method.

Outcome	Factor	Interaction driver and effect on deltas
*Vertical Shift*	BMI	*P* = 0.034 BMI impacts kV/kV method more than SGRT; increasing BMI leads to increasing vertical shift detected with kV/kV method
	AGE	*P* = 0.035 Age impacts kV/kV method more than SGRT; increasing age leads to increasing vertical shift detected with kV/kV method
*Roll*	Location	*P* = 0.065 Having a nonlung target location impacts SGRT more than kV/kV method.
	BMI	*P* = 0.022 BMI impacts kV/kV method more than SGRT; increasing BMI leads to decreasing roll detected with kV/kV method
*Rotation*	ROM	*P* = 0.057 4D ROM impacts kV/kV method more than SGRT; increasing ROM leads to increasing rotation detected with kV/kV method

BMI, body mass index; SGRT, Surface‐guided radiation therapy.

Based on these initial results, the standard method for patient setup prior to SBRT became SGRT followed by CBCT and subsequent patient data was only included for quantification and correlation of intrafraction motion.

### 3.3 SGRT detection and quantification of intrafraction motion during SBRT

**FIG. 1:**
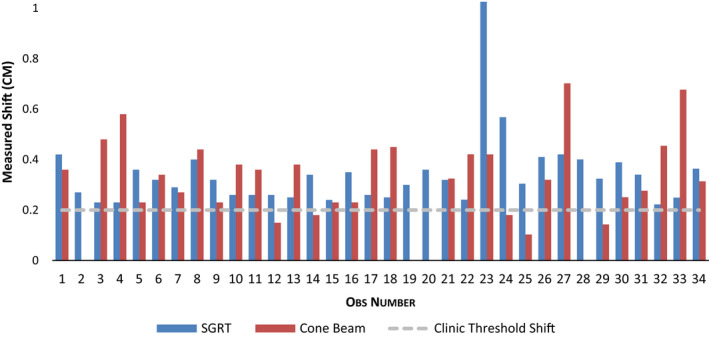
Vector intrafraction motion detected by SGRT (blue columns) and resulting CBCT shifts (red columns). CBCT, cone beam computed tomography; SGRT, Surface‐guided radiation therapy.

**FIG. 2:**
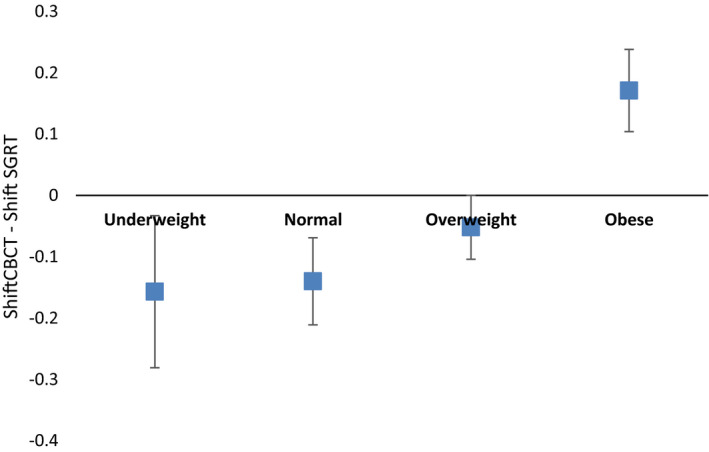
Difference in CBCT and SGRT Vector Shifts. CBCT, cone beam computed tomography; SGRT, Surface‐guided radiation therapy.

During the evaluation of intrafraction motion monitoring, there were 34 fractions for 25 unique patients where intrafraction motion was greater than the set tolerances described in the materials and methods section. This represented about 10% of the total fractions treated during the time period of data acquisition (total of 335 fractions). Table 4 shows results for detected intrafraction motion and resulting additional CBCT shifts on reimaging for all six degrees of freedom as well as the translational vector shifts. No statistically significant difference was found between the detected motion by SGRT and resulting CBCT shifts in the translational vector (paired t‐test *P* = 0.676). Additionally, the magnitudes of detected motion and resulting internal shifts in all six directions were observed to be numerically comparable. Of the 34 fractions where SGRT detected potential intrafraction motion of 2 mm or greater, repeat CBCT detected internal target motion of at least 2 mm in 25 or 73.5% of those fractions, and these resulting shifts were performed prior to continuation of treatment. The range of SGRT detected intrafraction motion and resulting CBCT shifts is illustrated in Fig. 1. Similar patient and tumor characteristics were analyzed to assess impact on correlation between SGRT detected motion and resulting internal vector shift target motion. Although age and BMI were univariately associated with the difference in shifts, only BMI remained significant after multivariable modeling (*P* = 0.019, Table 5). Figure 2 shows the effect of BMI on the difference in detected motion between SGRT and CBCT when BMI is separated into standard categories (underweight, normal, overweight, and obese). The estimated differences for underweight, normal, and overweight subjects indicate that, for each of these groups, SGRT on average overestimated the resulting shift on repeat CBCT. Conversely, the estimated difference for obese subjects indicates that, for this BMI group, SGRT on average underestimated the resulting shift on repeat CBCT.

**Table 4:** Detected intrafraction motion by SGRT and resulting additional CBCT shifts on reimaging. n = 25 unique patients (34 observations).

	*A*	*B*	*C*	D
	$\bar{\left|{\mathrm{Shift}}_{\mathrm{SGRT}}\right|}$	$\bar{\left|{\mathrm{Shift}}_{\mathrm{CBCT}}\right|}$	$\bar{\left({\mathrm{Shift}}_{\mathrm{CBCT}}-{\mathrm{Shift}}_{\mathrm{SGRT}}\right)}$	$\bar{\left|{\mathrm{Shift}}_{\mathrm{CBCT}}-{\mathrm{Shift}}_{\mathrm{SGRT}}\right|}$
Vertical (cm)	0.135	0.144	0.013	0.165
Longitudinal (cm)	0.196	0.109	−0.065	0.250
Lateral (cm)	0.100	0.158	0.016	0.169
Vector	0.332	0.314	−0.018	N/A
Pitch (˚)	0.376	0.112	−0.108	0.517
Roll (˚)	0.202	0.334	0.062	0.519
Rotation (˚)	0.286	0.038	0.143	0.309

A. This column contains the average of the magnitude of the shift deltas for the SGRT method, (calculated based on the absolute value of each shift). B. This column contains the average of the magnitude of the shift deltas for the Cone Beam method, (calculated based on the absolute value of each shift). C. This column is the average of the differences between CBCT measured shift and SGRT measured shift. D. This is the average of the magnitude (absolute value) of the differences between CBCT measured shift and SGRT measured shift.

CBCT, cone beam computed tomography; SGRT, Surface‐guided radiation therapy.

**Table 5:** Patient and tumor characteristics and correlation between SGRT detected motion and resulting internal target motion.

	$\left|{\mathrm{Shift}}_{\mathrm{CBCT}}-{\mathrm{Shift}}_{\mathrm{SGRT}}\right|$		$\left({\mathrm{Shift}}_{\mathrm{CBCT}}-{\mathrm{Shift}}_{\mathrm{SGRT}}\right)$			
	Univariable P‐value		Univariable association		Multivariable *P*‐value	
	Slope	*P*‐value	Slope	*P*‐value	Slope	*P*‐value
Gender (M vs F)	−0.007	0.900	−0.145	0.076	–	*–*
Site		0.612		0.941	–	*–*
Lung vs Other	0.053		0.038		–	*–*
Spine vs Other	−0.054		−0.007		–	*–*
Site Location		0.631		0.927	–	*–*
Peripheral vs N/A	0.063		0.036		–	*–*
Central vs N/A	0.082		0.057		–	*–*
Age	−0.003	0.346	−0.010	0.040	–	*–*
BMI	−0.0001	0.969	0.015	0.005	0.019	0.001
4D ROM	−0.074	0.301	−0.015	0.899	–	*–*
PTV Volume	−0.001	0.664	−0.0007	0.773	−0.254	0.049
ITV Volume	−0.002	0.391	−0.0003	0.954	0.041	0.088

BMI, body mass index; SGRT, Surface‐guided radiation therapy.

## 4 DISCUSSION AND CONCLUSIONS

The unique radiobiological characteristics of SBRT dose regimens are distinctly related to precise delivery of high dose per fraction treatment while minimizing organ at risk toxicity through careful planning and sharp dose falloff outside of the target volume. Dose delivery is inherently dependent on the quality of patient setup and verification of patient position both prior to and during treatment. While pretreatment volumetric imaging or “image guidance” has become a standard part of most commercially available linear accelerators, multiple systems have been developed to monitor the position of the target or patient during SBRT delivery. All of these systems use imaging of either the patient or the target during treatment. Imaging during SBRT treatment can involve additional equipment and image acquisition and quality can be limited by potential collisions with the patient or treatment table. In addition, the use of ionizing radiation for continuous imaging during treatment can significantly increase radiation exposure to the patient. Other systems that detect intrafraction motion by tracking a surrogate of the target typically require implanted fiducial markers or electromagnetic transponders that expose patients to invasive procedures that can be associated with serious complications, often in excess of the treatment itself.^18^ As described in AAPM Task Group 101, any localization and tracking method used during SBRT requires careful assessment of reliability and correlation.^2^

OSMS can detect submillimeter patient surface motion allowing for its use for several different treatment sites, including stereotactic treatments.^19^ While many studies have validated the accuracy of this system to track patient position during treatment, there have been few reports specifically attempting to analyze the effects of detected patient external motion on the resulting target displacement. Other studies have attempted to compare external to internal target position in the lung and suggest possible disparity, but few have evaluated a continuous external monitoring system such as SGRT. In one study looking at a SGRT system for SBRT treatments, patient position based on SGRT was compared to CBCT before treatment, but no additional monitoring was performed during treatment to correlate whether intrafraction motion detected by the SGRT system associated well with additional internal shifts on repeat CBCT.^20^ This study also suggested a good correlation between SGRT and interval CBCT for female patients but not for male patients. Our study did not find such a correlation based on a similar scaled analysis of patients.

The purpose of our study was to (a) quantify the reliability of SGRT in patient positioning prior to volumetric imaging to reduce interfraction patient positioning error and compare it to kV/kV imaging with bony anatomy match and (b) to measure the ability of SGRT to detect intrafraction patient motion that leads to target displacement during SBRT treatments. For the first part of our study, we compared the use of SGRT for patient positioning, which utilizes the patient’s surface as a reference, to one commonly used method of kV/kV imaging looking at bony anatomy in relation to the target prior to further volumetric imaging and target positioning with CBCT. While other studies have shown SGRT to be useful for initial positioning of a variety of conventionally fractionated treatment sites, ours is the first to evaluate SGRT for initial setup of SBRT patients.^21^, ^22^ Our results show that SGRT is an effective method for patient setup prior to SBRT treatments and is comparable to use of skin marks combined with kV imaging. This indicates that the 3D surface utilized by SGRT can be utilized prior to verification with CBCT. In addition, given the comparable reliability of SGRT with kV/kV match, additional imaging prior to CBCT is not necessary, reducing unnecessary radiation exposure in our clinic. Based on multivariate analysis, SGRT may provide less reliable initial setup for patients with higher BMI (>30), and therefore kV imaging prior to CBCT may be useful to reduce additional shifts for these patients.

In regard to intrafraction motion, SGRT detected even low quantities of patient intrafraction motion that resulted in additional shifts on CBCT. For the purposes of this study, 2 mm translational or 1 degree rotational shifts were determined to be translational threshold where the beam was held and the patient repositioned for the remaining portion of the treatment. While these thresholds are certainly smaller than our standard PTV margin, use of these thresholds proved important in that additional shifts up to 7 mm were made on repeat CBCT, indicating that SGRT can detect patient motion that may be a surrogate to detect target displacement that could be outside of PTV margin expansion for SBRT treatments in the lung. In addition, given the insignificant difference in the vector shifts detected by SGRT and the resulting adjustment made for patient position based on CBCT as shown by the paired t‐test, it could be reasoned that use of SGRT may facilitate the use of smaller PTV margin expansion for SBRT targets in these locations.

## 5 LIMITATIONS

There are some potential limitations to our study. With respect to target location, the majority of our patients had lung targets. Thus, inferences based on location may be difficult to assess based on small numbers outside of the thorax. It does appear that, similar to BMI, tracking the abdominal surface may affect the reliability of SGRT in comparison to other methods for initial setup. Target motion that is not detected by SGRT should also be considered as a possible limitation of this study. There were some shifts that were greater than what was detected by SGRT. We did have a “control” group where CBCT was repeated even though no motion was detected, and in 10 patients, no further adjustments were made on CBCT for 30 fractions. Another limitation of this study is that the dosimetric impact of the detected intrafraction motion is not described. In essence, does intrafraction motion of this quantity actually affect target coverage or organ at risk dose that would make it clinically meaningful? We have performed initial analysis of the dosimetric impact of the patients who had the greatest motion detected during treatment and the target dose and OAR dose was not affected. These data will be described in a separate manuscript. In addition, we intend to analyze the effect of detected intrafraction motion during lung and abdominal SBRT in relation to different PTV margin expansion quantities, which will allow accurate determination of what margin expansions are actually required during these highly precise treatments. PTV margin reduction for SBRT treatment in certain areas may allow for toxicity risk reduction and allow SBRT to be performed for tumors immediately adjacent to serial critical structures such as the esophagus, central airway, stomach, small bowel, duodenum, and central bile duct. These organs have all been shown to have maximum dose limiting toxicities with SBRT treatments to adjacent targets.

## 6 CONCLUSIONS

Based on our analysis, SGRT is a valuable tool in initial patient setup prior to CBCT and in detecting intrafraction patient motion during SBRT treatments that may allow for margin reduction without the use of ionizing radiation or invasive procedures. Due to the small margins and steep dose gradients needed for SBRT, our current standard practice is to utilize SGRT both for patient setup prior to volumetric imaging with CBCT and for intrafraction patient monitoring during treatment based on these results.

## CONFLICT OF INTEREST

John Heinzerling has received research funding from VisionRT, Inc for a project unrelated to this work. No other authors report a conflict of interest.
